# Editorial: Advanced invasive hemodynamics: pressure-volume maneuvers to obtain load-independent indices

**DOI:** 10.3389/fcvm.2024.1468811

**Published:** 2024-08-12

**Authors:** Filip Konecny, Sebastian Kelle, Alessio Alogna

**Affiliations:** ^1^ Department of Surgery, McMaster University, Hamilton, ON, Canada; ^2^ Schulich School of Medicine & Dentistry, Department of Medical Biophysics, Western University, London, ON, Canada; ^3^ Department of Cardiology, Angiology and Intensive Care Medicine, Deutsches Herzzentrum der Charité, Berlin, Germany; ^4^ Department of Cardiology, Angiology and Intensive Care Medicine, Deutsches Herzzentrum der Charité, Campus Virchow-Klinikum, Berlin, Germany; ^5^ DZHK (German Centre for Cardiovascular Research), Partner Site Berlin, Berlin, Germany

**Keywords:** hemodynamic, cardiovascular, catheterization, pressure-volume (PV) loops, preload and afterload responses

**Editorial on the Research Topic**
Advanced invasive hemodynamics: pressure-volume maneuvers to obtain load-independent indices

Hemodynamics, the study of the biophysical principles and laws controlling blood flow dynamics, has a long and rich history dating back several centuries. Over 350 years after Harvey's initial postulation of a blood circulation (1,628), in 1984 Baan and colleagues designed the first conductance catheter for the simultaneous and continuous acquisition of pressure-volume (PV) measurements in mammals (1). This breakthrough made it possible to perform more advanced hemodynamic evaluations in clinical settings. PV relationships are meanwhile broadly used in preclinical and clinical Cath-labs worldwide, to accurately assess cardiovascular function after various interventions, such as genetic, pharmacologic, or surgical manipulations. Nevertheless, while conductance catheters allow a deep understanding of cardiac (dys)-function, their current use in the daily Cath-lab routine can be difficult and necessitate a solid theoretical background and cautious interpretation. This research topic wants to provide relevant examples of standardized approaches in pressure-volume-flow analysis and assessment in different preclinical and clinical settings.

In the past decades modern therapy options such as mechanical circulatory support (MCS) devices are becoming increasingly popular, since more and more congestive heart failure patients are not responding to conventional medical therapies and face a relevant shortage of organ donors for transplantation. However, the potentially beneficial long-term effects of such devices on cardiac mechanics and structural remodeling still need to be accurately elucidated. Pamias-Lopez et al. summarize current evidence in the field, displaying elegant examples of left ventricular (LV) unloading induced by several MCS devices via PV diagrams, coupled to short- and long-term molecular mechanisms involved in beneficial structural remodeling observed in MCS patients.

Non-compressible truncal hemorrhage (NCTH) refers to severe bleeding in the torso area that cannot be controlled by external pressure or tourniquets and is a leading cause of potentially preventable deaths in trauma patients, particularly in military and civilian settings (2). In the context of life-saving interventions such as Resuscitative Endovascular Balloon Occlusion of the Aorta (REBOA) and Endovascular Variable Aortic Control (EVAC) for endovascular hemorrhage control, understanding PV dynamics is crucial to avoid irreversible myocardial injury following balloon inflation (3). Mobin et al. set out to investigate the variability in PV relationships during hemorrhagic shock and REBOA/EVAC interventions by leveraging on a previously published porcine hemorrhage model. They developed a novel algorithm to reliably quantify the single-beat and longitudinal P-V relations during hemorrhage and aortic occlusion. By continuous and accurate P-V loop assessment, they identified early hemodynamic predictors associated with declining cardiac performance during hemorrhage and REBOA use. This algorithm could help improve the management of an important and life-saving intervention in hemorrhagic shock.

Arterial stiffening and peripheral wave reflections have traditionally been considered primary factors influencing raised pulse pressure and isolated systolic hypertension. However, cardiac contractility and ventricular ejection dynamics are emerging as additional key factors in determining pulse pressure and its peripheral amplification (4). Piccoli and colleagues examined the relationship between pressure and aortic flow, as well as the relative contributions of arterial compliance and ventricular contractility to the observed changes upon pharmacological modulation *in vivo*, i.e., healthy volunteers and hypertensive patients, and in silico. Their findings demonstrate that changes in cardiac contractility alone can modify the shape of the forward pressure wave, leading to alterations in central and peripheral pulse phenotypes.

Coronary microvascular dysfunction (CMVD) after ST-elevation myocardial infarction (STEMI) affects approximately 30%–40% of STEMI patients, even after successful primary percutaneous coronary intervention, and it is of significant importance due to its impact on LV remodeling and patient outcomes (5). Severe microvascular dysfunction is the principal cause of microvascular obstruction (MVO), described as the underlying cause for the no-reflow phenomenon in STEMI patients. Wen et al. investigated the ability of the coronary angiography-derived index of microcirculatory resistance (CaIMR), calculated by combining computational flow and pressure simulation, to predict MVO, as assessed by cardiac magnetic resonance, in patients after STEMI (6). CaIMR combined with peak cardiac Troponin-I were found to be independent predictors of short-term MVO in patients with STEMI. This highlights the promising role of pressure and flow computation for understanding microcirculatory (dys)-function and obstruction.

In conclusion, the current research issue focused on invasive PV assessment of cardiovascular hemodynamics and showed its role in a variety of important clinical scenarios. From the impact of mechanical support devices on cardiac mechanics to the role of cardiac contractility in pulse pressure regulation, these findings provide valuable insights into the complex interaction between pressure, volume, flow and cardiac mechanics, reflecting the impact of several therapeutic approaches in cardiovascular medicine. As technology advances and methodologies evolve, pressure-volume analysis will continue to play a pivotal role in advancing our knowledge of cardiovascular physiology and pathology.
